# Advice and Information About Toothbrushing as Available on Websites of Professional Dental Care Associations

**DOI:** 10.1111/idh.70037

**Published:** 2026-03-23

**Authors:** Therese A. Elkerbout, Tim M. J. A. Thomassen, Fridus (G. A.) van der Weijden, Dagmar Else Slot

**Affiliations:** ^1^ Department of Periodontology Academic Centre for Dentistry Amsterdam (ACTA), a joint venture between the Faculty of Dentistry of the University of Amsterdam and the Faculty of Dentistry of the Vrije Universiteit Amsterdam Amsterdam the Netherlands

**Keywords:** brushing advice, brushing instructions, brushing techniques, dental care professional associations, dental hygienist association, dentist association, online, websites

## Abstract

**Aim:**

This study aimed to assess the online advice and information about toothbrushing provided by professional dental care associations (PDCAs) in English‐speaking countries.

**Material and Methods:**

PDCAs are national organisations of dentists and dental hygienists. A cross‐sectional internet search for information published until July 2021 was performed. First, it was assessed whether there was a PDCA in English‐speaking countries. A distinction was made between dentist associations (DAs) and dental hygienist associations (DHAs). In case a website was available, it was assessed whether toothbrushing advice was provided. Recommendations regarding a brushing technique, advice and instruction about the type of toothbrush, type of bristles, duration of brushing, time of changing the toothbrush, the use of toothpaste and interdental cleaning devices were gathered and summarised.

**Results:**

In total, 56 English‐speaking countries were considered, of which 52 had a DA and 15 had a DHA, the majority of which had a working website. In total, 35% of the PDCAs, that is, 16 DAs and 7 DHAs, provided advice and information on toothbrushing and recommended twice daily manual toothbrushing with a fluoride toothpaste. Generally, a toothbrushing duration of 2 min (*n* = 20) and the (modified) Bass toothbrushing technique (*n* = 9) were advised. Nine PDCAs recommended the use of a powered toothbrush as well, and 21 recommended the daily use of floss. Nine PDCAs also recommended the use of an interdental brush.

**Conclusion:**

In total, 35% of the PDCAs with a website provided advice and/or information on toothbrushing. All of the PDCAs recommended twice daily brushing with a manual toothbrush and fluoride toothpaste. However, a significant heterogeneity was observed in the instructions related to toothbrushing and toothbrushing technique. With respect to other recommendations on basic oral self‐care, there appears to be a need for alignment and improvement in professional information for the general public with respect to the type of toothbrush, toothbrushing instruction, technique and other toothbrushing‐related aspects.

## 1 Introduction

Periodontal diseases and dental caries are among the most common diseases in humans and the main causes of tooth loss. Patient cooperation in daily plaque removal is critical, as plaque is considered to be a major etiological factor [1, 2]. In public campaigns and communications regarding dental health, professional dental care associations (PDCAs) and governmental bodies recommend regular daily toothbrushing for preventing caries and periodontal diseases [3, 4, 5, 6]. Moreover, in oral care practice, dentists and dental hygienists commonly provide professional brushing instructions. Toothbrushing recommendations consist of information regarding not only the brushing technique, type of toothbrush, type of bristles, duration of brushing, and time of changing the toothbrush but also the use of toothpaste and the additional use of interdental cleaning. Available information must focus on individual patient needs and the personalised use of language that matches the patient's language level. This information can be provided verbally, with a brushing model, hands on, supplemented by the use of leaflets or via an app or web application [7, 8, 9].

PDCAs comprise dentist associations (DAs) and dental hygienist associations (DHAs). The World Dental Federation (FDI) [10] is considered an umbrella organisation as it unites the DAs and is the worldwide representative body for all dentists. It develops health policy, continuing education programs, serves as a unified voice for dentistry, and supports member associations in oral health promotion activities via the use of websites and social media. The International Federation of Dental Hygienists (IFDH) [11] has similar aims regarding dental hygienists and unites DHAs. PDCAs provide toothbrushing information on their websites. Patients with low health literacy are limited in the ability to find, understand, and use information and services to inform health‐related decisions and actions for themselves and others. Online information must adhere to professional standards as all information seekers view the credibility of the author or their affiliation as a key indicator of the quality of the content [12]. A low heterogeneity in the advice provided by different PDCAs may improve the patients' adherence to toothbrushing recommendations [13].

The aim of this study was to assess the advice and information about toothbrushing provided by PDCAs in English‐speaking countries as available on their websites.

## 2 Material and Methods

### 2.1 Context of Study

This report was prepared according to the STROBE guideline for cross‐sectional studies. This checklist recommends items that are included in the report [14, 15]. The collected data in this study were retrieved from publicly accessible websites. The Institutional Review Board of the Academic Centre for Dentistry Amsterdam (ACTA) approved the study protocol (#2020248).

Only PDCAs in English‐speaking countries were included in this study. Together, the USA, Ireland, and the 54 Commonwealth countries were included to represent the global context [16, 17]. The Commonwealth countries are united in a well‐known international organisation in connection with the United Kingdom (UK). Moreover, the UK government classifies the Commonwealth countries as well as Ireland and the USA as native English speaking [18, 19]. Altogether 56 countries were considered to be of interest.

### 2.2 Search Strategy for Professional Dental Care Associations (PDCAs)

First, a list of PDCAs in the 56 countries was made, with a distinction between DAs and DHAs. When a country had a DA or DHA, it was ascertained whether the association had a website. To locate the individual websites of these PCDAs, the search was primarily performed via the websites of the so‐called umbrella organisations—the FDI (World Dental Federation) [10] for the DAs and the IFDH (International Federation of Dental Hygienists) [11] for the DHAs. When no information was present or when information available was insufficient, the website www.suvison.com [20] was consulted, which provides global oral healthcare information on professional, technical, human, commercial and industrial resources online and offline. When no information was found, an individual search was performed using the search engine Google [21] because it has been shown to be effective in helping lay users obtain health and medical information [22]. The search terms used were: association, dental, dentist, dental hygienist, and the name of the corresponding country. To defeat the standard online search behaviour and to decrease the bias that occurs naturally in social networks as personalisation algorithms use increasingly more social data, the first 25 results of each search were considered and reviewed [23].

### 2.3 Data Extraction and Analysis

The content of the existing websites of DAs and DHAs in individual English‐speaking countries was explored for advice or information about aspects of toothbrushing. The websites were searched using their own search bar with the terms “brushing technique, advice and instruction”. When no search bar was available, the websites were searched using the Ctrl + F shortcut in the browser to find the words of interest, or a manual search was performed. Regarding available details, recommendations about the type of toothbrush, type of bristles, duration of brushing, time of changing the toothbrush, and use of toothpaste and interdental cleaning devices were extracted. All searches and data extraction were conducted in June 2021 independently by two examiners (TAE & TMJA).

Both DAs and DHAs and their related websites, if available, are listed in Appendix A in Supporting Information. The format (e.g., text, pictures, or videos) in which the available information on toothbrushing was provided was recorded. On the basis of the gathered information, a frequency table using absolute values and percentages was composed. In addition, for each country, the continent is specified, and it is indicated whether it is considered as a high‐, middle‐, or low‐income country [24].

## 3 Results

### 3.1 Included Countries and Websites

**FIGURE 1:**
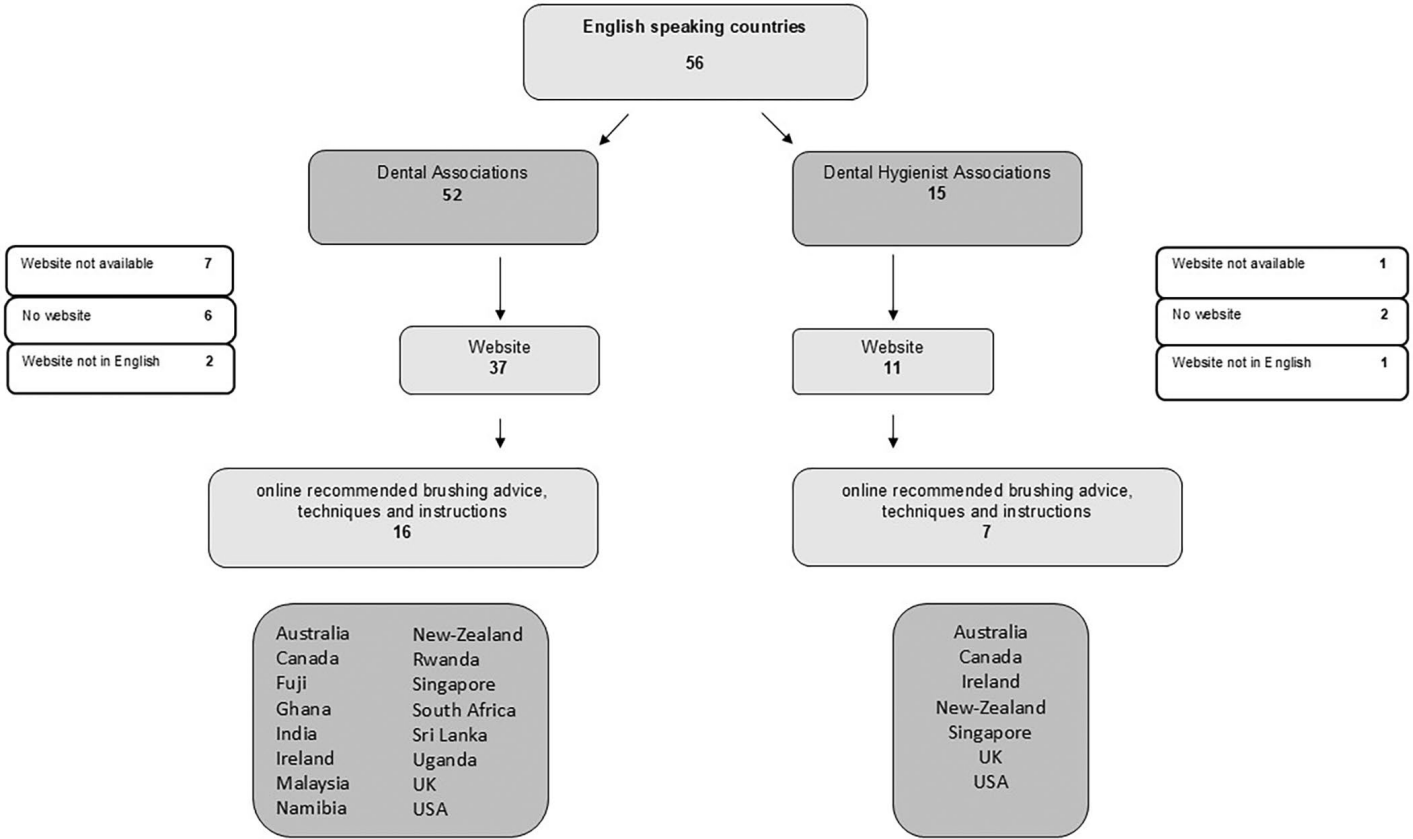
Flowchart of the online search for DAs and DHAs and their websites that provide online recommended brushing advice, techniques and instructions.

The online search showed that, of the 56 countries, 52 (92.6%) had a DA, and 15 (26.8%) had a DHA. For details of the included countries, see Figure 1 and Table 1.

**TABLE 1:** Outcome of the DAs and DHAs in the 56 countries and their association that provide online toothbrushing advice and information.

*N* = 56	DA	DHA
Countries with an association	52 (92.8%)	15 (26.8%)
Active website from associations	37 (71.2%)	11 (73.3%)
Online recommended toothbrushing information	16 (30.8%)	7 (46.7%)
Websites without toothbrushing information	21 (40.4%)	4 (26.7%)
Website not available	7 (13.4%)	1 (6.7%)
No website	6 (11.5%)	2 (13.3%)
Website not in English	2 (3.8%)	1 (6.7%)

*Note:* For details please see Appendix A in Supporting Information.

In four countries, the primary website found on the FDI website was not accessible. A Google search retrieved the dental council website of Mauritius and the Bahamas and the websites of the DAs of Rwanda and Sierra Leone. A website was found that was associated with Pakistan [25]. However, it was unclear which entity was responsible for the intellectual content; it appeared to have no connection with a DA or DHA. The website was therefore not considered for the present evaluation. The DHA website addresses found on the IFDH webpage for Singapore and South Africa turned out to be incorrect. A Google [21] search helped to retrieve the current web address. All data regarding the available DAs and DHAs and their corresponding websites are presented in the Appendix A in Supporting Information.

Two DAs (Cameroon and Cyprus) and one DHA (Cameroon) did not appear to have a website in English. Six countries had a DA but no website, and seven had a website that was not accessible (Appendix A in Supporting Information). The DHAs in the Bahamas and Pakistan also appeared to have no website, and the DHA of Jamaica had a website that was not accessible (Appendix A in Supporting Information). In total, 37 DAs (71.2%) and 11 DHAs (73.3%) had an online website in English (Appendix A in Supporting Information). Two DAs referred to specific affiliated consumer website MouthHealthy [26] (the USA) and the Oral Health Foundation [27] and the dental health section on the NHS website (the UK) [28]. In total, 16 (30.8%) DAs and 7 DHAs (46.7%) had websites that provide advice and information on toothbrushing (Table 1; Appendix A in Supporting Information). Only these 23 websites were suitable for inclusion in the present study as they provided structured advice and information on toothbrushing.

### 3.2 Brushing Advice, Techniques, and Instructions

Tables 2a and 2b present the details on toothbrushing advice and information provided on the included websites. All recommend the use of a fluoride toothpaste. All but two (Malaysia and Sri Lanka) recommended the additional use of dental floss. The advice and information on toothbrushing provided on DA and DHA websites regarding the type of toothbrush, type of bristles, duration of brushing, time of changing the toothbrush, and the use of toothpaste and interdental cleaning devices are categorised in Tables 2a and 2b, respectively. Most websites used text (*n* = 20) and videos (*n* = 13) to provide information concerning the toothbrushing method. All except one (South Africa) website gave the advice to brush twice daily and recommended 2 min or at least 2 min of brushing duration. Eleven websites emphasised that the time of day for brushing is advised to be morning and evening (or before bedtime). Brushing after every meal was recommended only by two DA websites (Canada and Rwanda).

**TABLE 2a:** Overview of the details given by the 16 DA association websites on toothbrushing information, by the format of the instruction method, details on toothbrushing and other extra advice and information.

DA per country *N* = 16	Format			Toothbrush														Other information				
				Method			Description			Moments			Type of brush		Replacement							
	Text	Pictures	Video	Technique	Frequency/day	Duration in minutes	Gently	Angle of 45°	Circular (C) Up‐and‐down (U) Back‐and‐forth (B)	Morning/night	After every meal	Before bedtime most important	Soft (S)/Medium (M)	Power TB	After x months	Bristles wear out	After being sick	Use fluoride TP	Spit not rinse	Clean/brush tongue	Daily use of floss	Daily use of IDB
Australia	+	+	+	MB	2	2	+	+	C	+			S	+	3	+	+	+	+		+	
Canada	+	+		MB	2	2–3	+	+	C/U		+	+	S		3			+			+	
Fiji			+		2	2												+			+	
Ghana	+				2	2							S/M		3–4			+	+		+	
India	+			MB	2	2	+	+	C	+		+	S	+	3–4	+		+		+	+	
				Fones^a^																		
Ireland	+	+		Scrub	2	2	Short sideway			+		+	M	+		+		+	+	+	+	
			+	MB			+	+	C													
Malaysia	+				2	2												+				
Namibia	+			MB	≥ 2		+	+	C			+	S	+				+		+	+	
New‐Zealand	+		+	Bass	2	2	+	+	B	+		+			3	+		+	+		+	+
Rwanda	+				≥ 2						+	+						+			+	+
Singapore	+		+		2	2	+	+	B									+			+	
South Africa	+				2								S	+				+		+	+	+
Sri Lanka	+				2	2–3	+	+	B	+		+	S	+				+		+		
Uganda	+		+		2	2									3		+	+			+	
UK	+		+		≥ 2	2		+	C	+		+	S	+	3^a^	+		+	+	+	+	+
USA (ADA)	+	+^a^	+		2	2	+	+	B				S		3–4	+		+		+	+	+
**Total**	**15**	**4**	**8**	**5 MB** **1Bass**	**16**	**13**	**9**	**10**	**6C** **4B** **1 U**	**6**	**2**	**8**	**9S** **2M**	**7**	**8**	**6**	**2**	**16**	**5**	**7**	**14**	**5**

^a^ Special instructions for children.

**TABLE 2b:** Overview of the details given by the 7 DHA association websites on toothbrushing information, by the format of the instruction method, details on toothbrushing and other extra advice and information.

DHA per country *N* = 7	Format			Brushing			Description			Moments			Type of brush		Replace			Extra information				
	Text	Pictures	Video	Technique	Frequency/day	Duration in minutes	Gently	Angle of 45°	Circular (C) Up‐and‐down (U) Back‐and‐forth (B)	Morning/night	After every meal	Before bedtime most important	Soft (S)/Medium (M)	Power TB	After x months	Bristles wear out	After being sick	Use fluoride TP	Spit not rinse	Clean/brush tongue	Daily use of floss	Daily use of IDB
Australia	+		+	MB	2	2				+			S		3	+	+	+	+	+	+	+
Canada	+	+	+	MB	2	2	+	+	U			+	S			+		+	+	+	+	
Ireland	+	+	+		2	2				+			S					+	+		+	+
New‐Zealand			+		2	2				+					3	+		+		+	+	
Singapore			+	Bass	2	2	+	+	C	+		+	S	+	3	+		+		+	+	+
UK	+	+			2	2	+	+	C	+		+	S/M	+	2–3	+		+	+	+	+	+
USA	+	+			2	2	+	+	B/C				S		3–4			+		+	+	
**Total**	**5**	**4**	**5**	**2 MB** **1Bass**	**7**	**7**	**4**	**4**	**3C** **1B** **1 U**	**5**		**3**	**6S** **1M**	**2**	**5**	**5**	**1**	**7**	**4**	**6**	**7**	**4**

The use of a powered toothbrush (PTB) was suggested as an alternative on nine websites. Regarding manual toothbrush features, a soft bristle configuration was advised on 15 websites. Only the DA of Ireland suggested a medium‐bristle brush, and two countries (Ghana [DA] and the UK [DHA]) recommended either a soft‐ or medium‐bristle toothbrush. The modified Bass brushing technique was mentioned on seven websites, and the Bass technique was mentioned twice [29]. Toothbrushing movements were mainly explained as circular followed by back‐and‐forth movements. Gentle brushing was specified on 13 websites (9 DA and 4 DHA) and positioning the manual toothbrush at an angle of 45° on 14 websites (10 DA and 4 DHA). It was advised on 13 websites (8 DA and 5 DHA) to replace the toothbrush after 2–4 months and on 11 websites (6 DA and 5 DHA) when bristle wear occurred. Replacement after being sick was advised by the PDCA of Uganda and by the DA and DHA of Australia.

### 3.3 Dentist Associations (DA) Compared With Dental Hygienist Associations (DHA)

In seven countries with a DHA website providing toothbrushing instructions, a DA website was also available. The use of a fluoride toothpaste, twice daily toothbrushing, and daily use of floss were common recommendations. In Australia, both the DA and DHA advised to replace the toothbrush after 3 months, when toothbrush bristles were worn, or after being sick. All other advice regarding toothbrushing was inconsistent between the DA and DHA websites within a country.

## 4 Discussion

### 4.1 Summary of Findings

In this cross‐sectional internet search study, the advice and information available online on websites of PDCAs in 56 English‐speaking countries were assessed. The focus was on oral hygiene instruction for public campaigns and communications regarding dental health with a primary emphasis on toothbrushing advice. Only 16 DAs and 7 DHAs provided online oral hygiene instructions for the general public on their website. The information on oral self‐care and the format in which it was provided were heterogeneous. There was a lack of consensus; however, there appeared to be universal agreement on the recommendation to brush twice daily with a fluoride‐containing toothpaste.

### 4.2 Locating Websites

The two main sources used to locate the targeted PDCA websites were the websites of the FDI and IFDH [10, 11]. If necessary, an additional Google search was performed [21]. To decrease search bias due to cookies and search engine algorithms, two independent researchers restricted their evaluation to the first 25 hits that emerged [23]. The decision of using only the first 25 hits was based on two earlier studies that have shown an overlap of 100% when different search engines and two or more reviewers were used [30, 31].

After the first 25 hits, it is most likely that the information found is not useful while performing a simple search with a few search terms in a search engine such as Google [21]. However, it cannot be ruled out that all available PDCA websites were not detected using this approach on Google. It is the responsibility of the website owners to ensure that their websites are easily found. A good search engine optimisation strategy helps the public find websites of their interest [32].

### 4.3 Professional Dental Care Associations (PDCAs)

The umbrella organisations FDI and IFDH both promote excellence in oral health, education, research, and practice and state that oral health is an integral aspect of overall health for both dental care professionals (DCPs) as well as the general public [10, 11]. In society, the internet is currently the main source of information; this also applies for the public in seeking patient‐related information. Communication via online platforms might be the method of choice to provide the public with dental health information [33]. It would be logical that public information on PDCA websites is based on clinical guidelines; it implies uniformity in advice and independent information. For example, there are various brushing techniques mentioned, different information about the brushing time of the day, and also the information about interdental cleaning is inconsistent. Based on the present findings, there appears to be a need for alignment and improvement in professional information for the general public with respect to toothbrushing. Umbrella PDCAs are in the best position to take responsibility for initiating and developing a basic guideline for self‐performed oral hygiene for the general public.

### 4.4 Format of Instructions

Online information about toothbrushing can be offered in different formats with text and/or visuals. The content format affects the process of information adaptation by the patient [7, 8, 9]. Visuals such as pictures, drawings, charts, graphs, and diagrams can be effective tools for providing information about health aspects, as this makes complex information easier to comprehend and more attractive [34]. Visuals also reinforce written or spoken health messages [34]. Meticulous toothbrushing is a procedure that requires more than only information adaptation; it also needs substantially higher cognitive performance to accomplish a behavioural change required to optimise dental hygiene self‐care [35].

For example, in a recent published randomised controlled trial among dental students [36], a brochure was provided that described the steps for learning a new toothbrushing technique. The results showed that no significant change in toothbrushing behaviour was seen, suggesting that providing only a brochure is insufficient to instruct and motivate even in a group of dental‐minded participants [36]. Alternatively, interest in the new toothbrushing technique may have subsided over time, or the lack of change may be related to the fact that the methods of toothbrushing recommended were either too difficult to perform or conflicted with what individuals such as patients had learned from other authorities [37, 38].

### 4.5 Recommendations

Two systematic reviews [39, 40] have shown that there is a lack of consensus on the recommendations for manual toothbrushing techniques both among professional dental (hygiene) organisations and in the scientific literature. There appears to be a wide diversity of recommendations [39, 40]. In addition, assessment of the effectiveness of dental health education indicates that there is room for improvement in the adherence to recommendations on aspects of toothbrushing [13]. This may suggest that there is limited effectiveness in the current public campaigns and communications regarding dental health. The currently available limited and varied information regarding toothbrushing from PDCA websites will presumably not improve the dental health of the general public.

Based on the present study it is recommended that umbrella PDCAs take responsibility to ensure alignment and consistency in advice on basic oral self‐care. This can be done by developing a guideline with evidence‐based recommendations about the type of toothbrush, brushing advice, technique, and other toothbrushing‐related instruction for the general public. The EFP guideline [1] could be used as a basis for evidence‐based advice and there is ample evidence available to support this from 4 meta‐reviews about the efficacy of mechanical cleaning [2], interdental cleaning [41], dentifrice [42] and mouthwash [43].

### 4.6 Barriers

All 56 included countries formally considered to be English speaking, but English is not the native language in all countries. In some of these countries, local languages are used in daily life, whereas English is the official language [44]. According to a systematic review on mobile health to improve the health behaviours and health outcomes of populations in developing countries, aspects such as language and literacy were found as important barriers. The analyses included Cameroon, Ghana, India, and Mozambique, all Commonwealth countries [45]. If websites are only provided in English, non‐native speakers are at a disadvantage, when the unofficial local language of native origin is more practical [45]. Canada, also a Commonwealth country [16, 17] is a good example of a country with two official languages, French and English. All governmental or PCDA websites are therefore bilingual which partly circumvents the disadvantage experienced by non‐native English speakers. Notably, Canada is a high‐income western society, and there are approximately 70 distinct indigenous languages belonging to 12 separate language families that are not used by official authorities or national organisations [46]. Regarding literacy, 70% of the countries included in the present study can be considered as developing and low‐income countries (Appendix A in Supporting Information). It is known that there is a correlation between income and education [47]. Thus, not only education but also other aspects such as poverty, race/ethnicity, age, and disability have an impact on health literacy [34, 48].

### 4.7 Strengths

An important strength of this study was that the 56 English‐speaking countries are extremely diverse and spread all over the world. They are among the largest, smallest, richest, and poorest countries [16, 17, 18, 19]. They include first‐, second‐, and even third‐world countries with a high to low income, which makes them a representative selection for this research. The inclusion of these countries reduces selection bias, making the results more representative of what can be universally expected. Another strength of this study was the availability of the two umbrella organisations FDI and IFDH [10, 11], a collective of DAs and DHAs, respectively, which provided a good starting point for the internet search strategy. However, some of the DAs and the DHAs could be found only via Suvison and Google [20, 21].

### 4.8 Limitations

In addition to PDCAs, dental councils, dental chambers, or dental unions may also be present in some countries, with an overlap in their objectives. In the present study, only those organisations aiming to improve the oral health of the general public were targeted. Consequently, unions for the advocacy of DCPs were not considered as eligible.

Some countries consider dental hygienists and dental therapists as distinct professions. Based on its origin, dental hygienists have a preventive and care focus and dental therapists have a treatment and cure focus. The professional profile of a dental therapist does not overlap that of a dentist or a dental hygienist [49]. However, in some countries such as the UK and Singapore, dental hygienists and dental therapists are united into a single profession [50, 51, 52]. Nevertheless, dental therapists as a distinct profession were not taken into account in the present study. Due to all the different names and allocated activities in the mouth as dental care professionals, this research limited the inclusion only for the DA and DHA associations.

### 4.9 Recommendation for Further Research

The information and advice given by PDCA's can be extended to healthy mouth parameters and disease information on caries, gingivitis and periodontitis. In addition this can be correlated to the oral health situation in the respective countries and also related to the number of dentists and dental hygienists/dental therapists. The global health status of the WHO can be used as a source for this evaluation [53].

## 5 Conclusion

The majority of the 56 English‐speaking countries had a PDCA; 52 countries had a DA, 15 of which also had a DHA. In total, 35% of the PDCAs with a website provided advice and/or information on toothbrushing. All of the PDCAs recommended twice daily brushing with a manual toothbrush and fluoride toothpaste. However, a significant heterogeneity was observed in the instructions related to toothbrushing and toothbrushing technique. With respect to other recommendations on basic oral self‐care, there appears to be a need for alignment and improvement in professional information for the general public with respect to the type of toothbrush, toothbrushing instruction, technique and other toothbrushing‐related aspects.

## 6 Clinical Relevance

### 6.1 Scientific Rationale

Professional dental care associations (PDCAs) are national organisations of dentists and dental hygienists. It is of interest what these professional organisations communicate to the general public about oral care.

### 6.2 Principal Findings

Not all 67 PDCAs (52 DAs and 15 DHAs) had a website, and some with a website did not provide advice or information about toothbrushing. Twice daily toothbrushing with a manual brush and a fluoride toothpaste was recommended by 16 DAs and 7 DHAs on their websites.

### 6.3 Practical Implications

There appears to be a need for alignment and improvement in professional information for the general public with respect to the type of toothbrush, toothbrushing instruction, technique, and other toothbrushing‐related aspects. Umbrella PDCAs are in the best position to take responsibility for initiating and developing a universal guideline for the general public.

## Author Contributions

All authors gave final approval and agreed to be accountable for all aspects of work, ensuring integrity and accuracy. **Therese A. Elkerbout:** contributed to design, analysis and interpretation, and drafted the manuscript. **Tim M. J. A. Thomassen:** contributed to analysis and interpretation and critically revised the manuscript. **Fridus (G. A.) van der Weijden:** contributed to analysis and interpretation and critically revised the manuscript. **Dagmar Else Slot:** contributed to conception and design, analysis and interpretation, and critically revised the manuscript.

## Funding

The authors have nothing to report.

## Ethics Statement

The protocol was registered at the ACTA (#2020248).

## Conflicts of Interest

The authors declare no conflicts of interest. The work for this paper was funded by the regular academic appointment of Slot and van der Weijden at the Academic Centre for Dentistry Amsterdam (ACTA). Slot and van der Weijden have previously received either external advisor fees, lecturer fees, or research grants from toothbrush manufacturers. Those manufacturers included Colgate, Dentaid, GABA, GSK, Lactona, Oral‐B, Procter & Gamble, Philips, Sara Lee, Sunstar, Waterpik, and Unilever. van der Weijden has formerly received two unrestricted educational grants from Procter & Gamble Worldwide Clinical Investigations—Oral Care.

## Supporting information


**Data S1:** idh70037‐sup‐0001‐DataS1.pdf.

## Data Availability

The data that support the findings of this study are available from the corresponding author upon reasonable request.
