# Supplementary material for: Advice and Information About Toothbrushing as Available on Websites of Professional Dental Care Associations
Source: Int J Dent Hyg. 2026 Mar 23;24(3):472–80. doi: 10.1111/idh.70037 (PMC13309212; doi:10.1111/idh.70037)
Supplement: Supplementary file 1 — Data S1: idh70037‐sup‐0001‐DataS1.pdf. [file IDH-24-472-s001.pdf]

Advice and Information about toothbrushing as available on websites of professional dental care associations

Therese A. Elkerbout

Tim MJA Thomassen

Fridus (GA) van der Weijden

Dagmar Else Slot

Appendix

Appendix A

Overview of all 56 countries presenting a DA and DHA and their association websites including whether they provide toothbrushing information

Country	Continent	Income level	DA	Association website	Brushing Information	DHA	Association Website	Brushing Information
Antigua and Barbuda*	North-America	H	-			-		
Australia*	Oceania	H	+	Australian Dental Association https://www.ada.org.au	++	+	Dental Hygienist Association of Australia https://www.dhaa.info ***	++
The Bahamas*/*****	North-America	H	+	Bahamas Dental Council www.bahamasdentalcouncil.com	□	+	**No website**	
Bangladesh*	Asia	L/M	+	http://www.bangladeshdentalsociety.org	□	-		
Barbados*	North-America	H	+	http://www.barbadosda.org **>> NA**		-		
Belize*****	North-America	L/M	+	https://www.belizemedicaldentalassociation.com/	□	-		
Botswana**/*****	Africa	U/M	+	www.bodeabw.com **>>NA**	□	-		
Brunei**	Asia	H	-			-		
Cameroon**/*****	Africa	L/M	+	Website in French		+	Website in French	
Canada*	North-America	H	+	Canadian Dental Association https://www.cda-adc.ca/	++	+	Canadian Dental Hygienist Association	++

							https://cdha.ca *** https://www.dentalhygienecanada.ca	
Cyprus*	Asia	H	+	Website in Greek		-		
Dominican Republic**	North-America	U/M	+	www.asociacionodontologicadominicana.com **>> NA**		-		
Eswatini/Swaziland*****	Africa	L/M	+	https://eswatinimedicalcouncil.com/	□	-		
Fiji*****	Oceania	U/M	+	Fiji Dental Association https://fijida.org/	++	-		
Gambia**	Africa	L	+	http://gmda.gm **>> NA**		-		
Ghana*	Africa	L/M	+	Africa Ghana Dental Association https://www.gdaonline.org/	++	-		
Grenada*****	North-America	U/M	+	https://gmdc.gd/	□	-		
Guyana*****	South-America	U/M	+	https://dpi.gov.gy/tag/guyana-dental-association/	□	-		
India*	Asia	L/M	+	Indian Dental Association https://ida.org.in/	++	+	https://fidh.in***	□
Ireland*	Europe	H	+	Irish Dental Association https://www.dentist.ie	++	+	Irish Dental Hygienists Association https://www.idha.ie ***	++

Jamaica**	North-America	U/M	+	https://jamaicadentalassociation.org/	□	+	https://www.dhaj.org/***** **>>NA**	
Kenya*	Africa	L/M	+	www.kda.or.ke/	□	-		
Kiribati**	Oceania	L/M	+	**No website**		-		
Lesotho*****	Africa	L/M	+	www.lmc.org.ls	□	-		
Malawi**/*****	Africa	L	+	**No website**		-		
Malaysia*	Asia	U/M	+	Malaysian Dental Association https://mda.org.my/	++	-		
Maldives**	Asia	U/M	+	http://mmc.gov.mv/	□	-		
Malta*	Europe	H	+	www.dam.com.mt	□	+	https://maltahygienists.com/*****	□
Mauritius*/*****	Africa	U/M	+	http://mauritiusdentalassociation.mu **>> NA** www.dentalcouncilmu.org www.adpmauritis.tripod.com	□	-		
Mozambique*	Africa	L	+	www.ammd-mz.com**>> NA**		-		
Namibia**	Africa	U/M	+	Namibian Dental Association http://namibiadent.com/	++	-		
Nauru**	Oceania	H	+	**No website**		-		

New Zealand*	Oceania	H	+	New Zealand Dental Association https://www.nzda.org.nz/	++	+	Dental Council of New Zealand: www.dcnz.org.nz ***	++
Nigeria*	Africa	L/M	+	https://nigdentalasso.org/	□	+	https://dthbn.gov.ng/*****	□
Pakistan*	Asia	L/M	+	http://www.pda.org.pk	□	+	**No website**	
Papua New Guinea**	Oceania	L/M	+	**No website**		-		
Rwanda*/*****	Africa	L	+	Rwanda Dental Association http://rda.rw/ http://rwandadentalassociation.com/ > NA	++□	-		
Saint Kitts and Nevis*****	North-America	H	+	https://www.sknmda.org/	□	-		
Saint Lucia*****	North-America	U/M	+	http://www.slmda.org/	□	-		
Saint Vincent and the Grenadines*****	North-America	U/M	+	www.health.gov.vc	□	-		
Samoa**	Oceania	L/M	+	**No website**		-		
Seychelles*****	Africa	H	+	https://s-mdc.org/	□	-		
Sierra Leone**/*****	Africa	L	+	http://www.slmda.sl > NA www.mdc-sl.org	□	-		
Singapore*	Asia	H	+	Singapore Dental Association http://www.sda.org.sg	++	+	Association of Oral Health	++

							Therapists<br>https://www.aoht.org.sg/ ****	
Solomon Islands**	Oceania	L/M	-			-		
South Africa*	Africa	U/M	+	The South African Dental Association<br>https://www.sada.co.za/	++	+	www.ohasa.co.za ****	□
Sri Lanka*	Asia	L/M	+	Sri Lanka Dental Association<br>http://www.slda.lk/	++	-		
Tanzania*	Africa	L/M	+	http://www.tdadent.or.tz	□	-		
Tonga**	Oceania	U/M	+	**No website**		-		
Trinidad and Tobago*****	North-America	H	+	https://dctt.org.tt/site/home.php	□	-		
Tuvalu	Oceania	U/M	-			-		
Uganda*	Africa	L	+	Uganda Dental Association<br>http://www.ugadent.org/	++	-		
United Kingdom*	Europe	H	+	British Dental Association<br>https://bda.org<br>Refer to:<br>https://dentalhealth.org<br>www.nhs.uk	++	+	British Society of Dental Hygiene and Therapy<br>http://www.bsdht.org.uk/ ***	++

USA*	North-America	H	**+**	American Dental Association www.ada.org Refer to: https://www.mouthhealthy.org/en	**++**	**+**	American Dental Hygienists Association http://adha.org ***	**++**
Vanuatu	Oceania	L/M	**+**	http://www.vda.vu**>>NA**		**-**		
Zambia	Africa	L/M	**+**	http://www.medicstravel.co.uk/countryhospitals/africa/zambia.htm **>>NA**		**-**		

+ = a DA or DHA available - = no DA or DHA available NA= website not working/not available

++ = brushing advice, techniques and instructions recommended □ = no brushing advice, techniques and instructions recommended

H= high Income level U/M= Upper/Middle income level L/M= Low/Middle income level L=Low income level

*website found through search on FDI-member list

** website found through search on Suvison-DA list

*** website found through search on IFDH-member list

**** website not correct on the IFDH-member list

***** website found on Google
